# Hide-and-Seek: A Game Played between Parasitic Protists and Their Hosts

**DOI:** 10.3390/microorganisms9122434

**Published:** 2021-11-25

**Authors:** Iva Kolářová, Andrea Valigurová

**Affiliations:** 1 Laboratory of Vector Biology, Department of Parasitology, Faculty of Science, Charles University, Albertov 6, 128 44 Prague, Czech Republic; 2 Department of Botany and Zoology, Faculty of Science, Masaryk University, Kotlářská 2, 611 37 Brno, Czech Republic

**Keywords:** unicellular parasite, parasitic protist, *Cryptosporidium*, *Leishmania*, intracellular, epicellular, extracellular, parasitophorous sac, parasitophorous vacuole, adaptation to parasitism, evasion strategies, host defence

## Abstract

After invading the host organism, a battle occurs between the parasitic protists and the host’s immune system, the result of which determines not only whether and how well the host survives and recovers, but also the fate of the parasite itself. The exact weaponry of this battle depends, among others, on the parasite localisation. While some parasitic protists do not invade the host cell at all (extracellular parasites), others have developed successful intracellular lifestyles (intracellular parasites) or attack only the surface of the host cell (epicellular parasites). Epicellular and intracellular protist parasites have developed various mechanisms to hijack host cell functions to escape cellular defences and immune responses, and, finally, to gain access to host nutrients. They use various evasion tactics to secure the tight contact with the host cell and the direct nutrient supply. This review focuses on the adaptations and evasion strategies of parasitic protists on the example of two very successful parasites of medical significance, *Cryptosporidium* and *Leishmania*, while discussing different localisation (epicellular vs. intracellular) with respect to the host cell.

## 1. Introduction

Hide-and-seek is a popular children’s game played all over the globe. The very same game is also played between parasites and their hosts. On one side of this game, there is a parasite trying to use the host’s resources to produce progeny. On the other side, there is a host which defends its internal resources with the help of the immune system. The host selects the most effective weapons according to the parasite localisation.

Extracellular parasites develop and multiply at the host extracellular sites such as mucosal surfaces, interstitial spaces, and body fluids. In the gut lumen, extracellular parasites (e.g., *Giardia intestinalis*, syn. *G. lamblia* or *G. duodenalis*) have to deal with physiological processes of digestion as well as with antimicrobial peptides and IgA antibodies [1]. In the blood, extracellular parasites (e.g., *Trypanosoma brucei*) have to evade the complement system, phagocytes, and later also specific antibodies [1].

To survive in such a hostile environment, some parasites adapted to hide inside the host cell. Parasitic protists developed two different strategies to hide themselves from the extracellular environment: intracellular and epicellular parasitism. 

Intracellular parasites (e.g., *Leishmania* spp., *Trypanosoma cruzi*, *Toxoplasma gondii*) are able to enter the host cell below the plasma membrane and survive or even multiply there; obligate intracellular parasites cannot multiply extracellularly [1]. Parasites can enter the host cell by a passive or an active process. The exact entry strategy depends on the parasite species and the host cell type. Inside the host cell, parasites are localised either freely in the cytoplasm or surrounded by a membranous compartment. Such compartment is usually of the host cell origin (e.g., endosome, phagosome, phagolysosome) modified by the parasite-derived molecules and thus called a parasitophorous vacuole [1].

Epicellular parasites (e.g., *Cryptosporidium* spp. and some eimeriid apicomplexans from coldblooded hosts) are somewhere in a transition state between extracellular and intracellular parasitism. These pathogens are bound to the cell surface, some even affecting the architecture of the host cell [2]. While the epicellular localisation is well defined for bacteria, the definition of epicellularity in parasitic protists is still a matter of a scientific debate [3]. For the purpose of this review, we define the epicellular parasitism in protists as being bounded to the host cell surface, above the host cell plasma membrane. These parasites do not penetrate the plasma membrane of the host cell and do not enter its cytoplasm [3,4,5]. Although they are localised above the plasma membrane, epicellular parasites are tightly bound to the host cell surface and directly interact and modify the host’s cell intrinsic processes. Some of these epicellular parasites are partially or completely surrounded by a membranous compartment called the parasitophorous sac that emerges from the plasma membrane, covering the host cell apical surface [3].

Neither of the two niches (intracellular and epicellular) are, however, completely safe and parasites still have to protect themselves against host cell defence mechanisms and immune cells that are in search of infected cells.

This review focuses on adaptations to epicellular and intracellular parasitism in the context of parasite evasion strategies. We will discuss these two types of parasitism using primarily *Cryptosporidium* and *Leishmania* parasites as an example, respectively.

## 2. Neither Naked nor Clothed: A Smart Strategy for Epicellular Parasitism in Cryptosporidia

Intracellular localisation protects the parasitic protists from the significant number of immune system weapons (e.g., antibodies, complement system, and phagocytosis). On the other hand, intracellular parasites must face the host cell repair and defence mechanisms. To keep the advantage of intracellular localisation and—at the same time—to avoid cellular defence mechanisms, some parasites came up with a clever solution of epicellular parasitism, mastered in *Cryptosporidium* and some eimeriids (Apicomplexa) from poikilotherm hosts. 

### 2.1. Formation of Parasitophorous Sac

**Figure 1:**
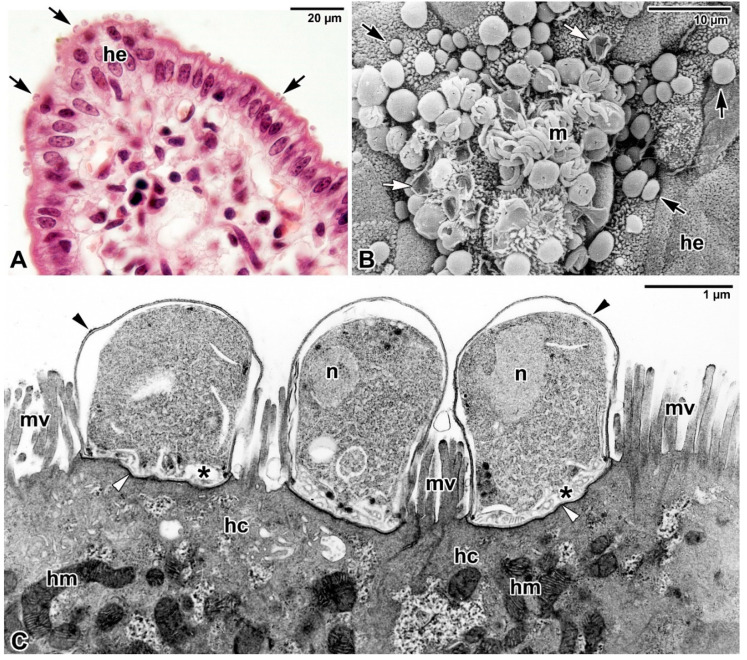
Epicellular localisation of *Cryptosporidium parvum* parasitising the intestinal epithelium of BALB/c mice. (**A**) Histological section showing the ileal villus parasitised by various developmental stages of *C. parvum* (black arrows). *he*—host intestinal epithelium. Haematoxylin-eosin staining, Light microscopy. (**B**) The luminal surface of colon heavily parasitised by various developmental stages of *C. parvum* (black arrows) including the exposed merozoites (m). White arrows mark empty parasitophorous sacs (PS). *he*—host intestinal epithelium. Scanning electron microscopy. (**C**) Three trophozoites of *C. parvum* attached to the host ileum and completely enveloped by the PS (black arrowhead). Note the dense band separating the modified part of the host cell (=PS) from the unmodified part (white arrowhead) with a fully developed feeder organelle (asterisk) at the parasite-host interface. *hc*—host cell, *hm*—host mitochondria, *mv*—host microvilli, *n*—parasite nucleus. Transmission electron microscopy. Electron micrographs (**B**,**C**) courtesy of prof. Břetislav Koudela.

*Cryptosporidium* invasive stages (sporozoites, merozoites) are polarised cells with characteristic organisation of apicomplexan zoites [6,7]. They prefer to enter and develop within the epithelial brush border of [3,4,8,9] of the gastrointestinal and respiratory tracts in a wide range of vertebrate hosts, including humans. Within the microvillous layer of epithelial cells, cryptosporidia reside in a peculiar niche—while some refer to them as intracellular extracytoplasmic parasites [10], others prefer the term epicellular to reflect their unique location in a host-derived parasitophorous sac (PS) more accurately [3,4,9,11,12,13,14,15,16,17,18]. Detailed electron microscopic observations support the term epicellular as *Cryptosporidium* invasive stages neither penetrate under the host cell plasma membrane nor come into the close contact with the host cell cytoplasm [3,4]. The invading parasite, however, induces significant reorganisation and remodelling of the host cell actin. *Cryptosporidium* employs the host actin-binding protein villin to reorganise and stabilise F-actin bundles within host cell microvilli, and the actin-cross-linking protein α-actinin to assembly individual filaments into an actin plaque at the host-parasite interface [19]. This results in significant modification of the host cell plasma membrane losing its microvillous character and transforming into a circular fold that gradually encapsulates the parasite attached to the apical surface of the host cell, and finally forms PS (Figure 1).

The accumulation of host cell actin starts at a point just below the attachment of the parasite, but later it has a character of an annular plaque underlying the parasite attachment site and, in a basket-like pattern, extending in the folds of the cell membrane, forming the PS (with increased accumulation in its basal region, but significantly decreasing posteriorly) [18,20]. This actin plaque accumulating at the parasite attachment site appears as a dense band formed by microfibrils interwoven perpendicularly with an adjacent network of actin filaments and separating the modified and unmodified parts of the parasitised cell. The dense band is considered to function in firm parasite anchoring and appears to prevent the penetration of the parasite into the host cell cytoplasm [3,21,22,23,24]. The rate of host cell plasma membrane protrusion depends on both the rate of actin polymerisation and the increase in localised cell volume, with glucose-driven and aquaporin-mediated localised water influx involved in this membrane protrusion during *Cryptosporidium* invasion [24]. As the gliding motility of apicomplexan zoites is generally considered the main mechanism facilitating host cell invasion, of particular interest is the simulated in vitro infection of human cell lines (HCT-8 and HT29) with polystyrene microspheres coated with *C. proliferans* homogenate, which has revealed that parasite encapsulation by cultured cells is induced only by parasite antigens, independent of any active invasion or gliding motility [14].

**Figure 2:**
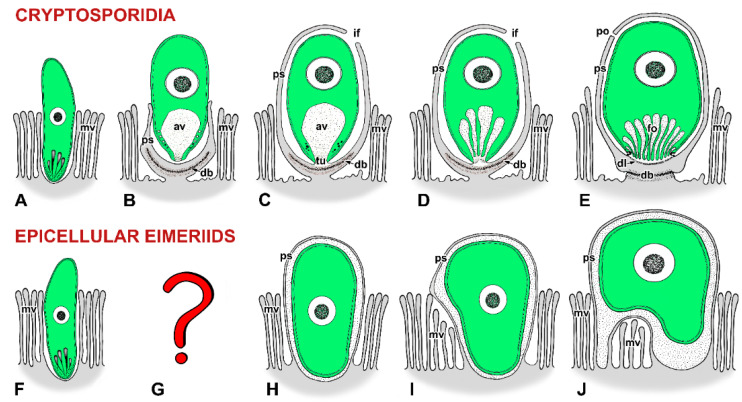
Schematic diagram of host-parasite interactions in cryptosporidia (**A**–**E**) and epicellular eimeriids (**F**–**J**). Three colours are used to distinguish between the parasite (in green), the host cell including its parts modified due to parasitisation (in grey), and the contact zone between the host and the parasite (in white with black dots) where the interactions of the two organisms become more intimate. (**A**–**E**) Cryptosporidia: (**A**) Invading zoite (either sporozoite or merozoite). (**B**) An early trophozoite partially enveloped by an incomplete parasitophorous sac (PS). (**C**) Young trophozoite almost completely enveloped by a PS. Note the tunnel connection between the interior of the anterior vacuole and the host cell cytoplasm that developed as the result of the Y-shaped membrane junction. (**D**) Almost mature trophozoite. Note the folding of the anterior vacuolar membrane during its transformation into the feeder organelle. (**E**) Mature stage with a prominent filamentous projection at the base of the PS and with a fully developed feeder organelle, the lamellae of the feeder organelle formed from the anterior vacuole membrane. (**F**–**J**) Epicellular eimeriids: (**F**) Invading zoite. (**G**) The transformation process of the zoite into an early trophozoite stage has not been documented. (**H**) A trophozoite enveloped by a PS with a single attachment area (monopodial form). (**I**) Extension of the later developmental stage above the microvillous region leading to an establishment of a new contact with the host cell apart from the primary attachment zone. (**J**) The spider-like form with several areas of the PS contacting the apical surface of host enterocytes. It may overlap two or more enterocytes. *av*—anterior vacuole, *db*—dense band, *dl*—dense line separating the feeder organelle from the filamentous projection of the PS, *fo*—feeder organelle with membranous lamellae, *if*—incomplete fusion of PS, *mv*—host microvilli, *po*—pore on the PS, *ps*—parasitophorous sac, *tu*—tunnel connection.

While the exact mechanism of nutrient uptake by *Cryptosporidium* is not clear, it is believed that PS has a protective role and the feeder organelle is a site regulating the transport of nutrients—and consequently also drugs during treatment—to the parasite [4,25,26]. A similar parasitisation strategy can be found in much less studied apicomplexans—eimeriids from coldblooded vertebrates (e.g., *Choleoeimeria*, *Acroeimeria*, some *Goussia*, and *Eimeria* formerly known as *Epieimeria*) and protococcidian *Eleutheroschizon duboscqi* from marine polychaetes [5,27,28,29,30,31,32]. All these apicomplexans invade the brush border in the host’s gastrointestinal tract and stimulate additional growth and fusion of host cell microvilli accompanied by modification of host cell plasma membrane, leading to the gradual PS formation (Figure 2). In some epicellular eimeriids (e.g., *Goussia janae* from a fish host) additional microvilli from several enterocytes may be added gradually and fused to enlarge the epicellular envelope, resulting in formation of the spider-like forms in older developmental stages (Figure 2I,J). Either way, this PS provides a suitable niche for the parasite to develop in the cavity of a host-derived capsule, which separates it from the lumen of the host gastrointestinal tract. Although there are some differences in the attachment strategies between cryptosporidia and epicellular eimeriids, e.g., not complete PS in cryptosporidia connecting them directly to the host cell via the feeder organelle (Figure 2E) vs. the inner membrane of the PS enveloping the entire parasite in *E. duboscqi* and eimeriids (Figure 2J), the goal of this peculiar niche might be the same—to more effectively avoid the host defence responses [5,30].

### 2.2. Parasite Invasive Apparatus and Its Role in Modulation of Host Cell Actin

*Cryptosporidium* is well adapted to this epicellular localisation. In contrast to apicomplexan parasites with intracellular localisation (e.g., *Toxoplasma gondii*, *Plasmodium* spp.), it lacks molecules important for the host cell invasion and the formation of parasitophorous vacuole (PV). In general, apicomplexan parasites are characterised by the presence of the apical complex, usually consisting of a cytoskeletal backbone (conoid, apical polar ring(s), spirally arranged subpellicular microtubules) and secretory membrane-bounded organelles (rhoptries, micronemes, dense bodies), which plays an important role in host cell invasion. In intracellular apicomplexans developing within PV, the rhoptry proteins are injected into host cell cytoplasm, and the core rhoptry neck proteins (RONs) complex inserted into the host plasma membrane interacts with the apical membrane antigen 1 previously secreted by the parasite micronemes and anchored to its plasma membrane. This results in a tight and irreversible connection (moving junction) between the invading parasite and the host cell plasma membrane, which drives the invasion by moving from the apical to the posterior end of the parasite, leading to the parasite internalisation within PV. Rhoptry bulb proteins (ROPs) and dense granules proteins act by redistribution and remodelling of the composition of the PV membrane, so that it does not fuse with the host cell endolysosomal system and allows the intracellular parasite to access host metabolites [33,34]. Some rhoptry proteins are even thought to subvert host cell functions and modulate immune response [34]. 

While *Cryptosporidium* possesses the very same secretory organelles of the apical complex—micronemes, rhoptries and dense granules—it lacks the molecular components of the moving junction [30,35], fitting perfectly to the ultrastructural evidence that cryptosporidia do not form this invasion device of intracellular parasites [18]. Instead, the proteins secreted by *Cryptosporidium* apical organelles are tailored to create the epicellular niche. They mediate the parasite attachment and orchestrate the formation of the parasitophorous sac and the feeder organelle. Micronemal proteins in *C. parvum* are located in the interior space of the PS but not in host cell cytoplasm [36], indicating the only region of host cell modified by microneme secretion during parasite invasion is the protruding apical host plasma membrane. Moreover, in contrast to intracellular parasites, *Cryptosporidium* sporozoites are equipped with a single rhoptry with a poorly known repertoire of molecules [18,20,37]. The presence of only one organelle, which is considered essential for the successful apicomplexan invasion, may result in the parasite having only a single attempt for a successful attachment to the host cell [37] but only if it also plays a significant role during formation of epicellular PS. The membranous content of this single rhoptry appears to form the numerous lamellae (folds) of the feeder organelle, corresponding to the parasite attachment site and separating the host and the parasite cytoplasm [4,33,38]. From six *C. parvum* ROPs identified so far, ROP2 and ROP4 localise exclusively to the PS (most likely in its interior space), ROP5 and ROP6 localise in a ring at the host-parasite interface parallel to the host epithelium, and ROP1 and ROP3 localise in both the PS and in the host cell (ROP3 evenly distributed in the parasitised cell, ROP1 accumulated in apical periphery of host cell rich in actin) [20]. A single *C. parvum* protein (CpPRP1), shown to be homologous to known RONs, also appears to be involved in host cell actin recruitment at the site of parasite attachment [18]. In conclusion, while the number of RONs conserved among species is generally involved in host cell invasion by intracellular apicomplexans, less well-conserved ROPs likely evolved for a particular lifestyle [33], and in cryptosporidia the effect of discovered rhoptry proteins appears to be more focused on modulating host cell actin. 

Actin dynamics are crucial for maintaining the epithelial cell junction, a barrier that prevents many pathogens from crossing the mucosal epithelium and spreading in the host body. The unique way by which *Cryptosporidium* parasitises manipulates host epithelial cells is particularly interesting but remains to be fully elucidated. Recently discovered ROP1 was found to bind host protein LIM domain only 7 (LMO7), an organiser of epithelial cell polarity and cell-cell adhesion, and accumulate at the terminal actin web, located just below the brush border of mice enterocytes. Genetic ablation of LMO7 and ROP1 in mice and parasites, respectively, suggest that LMO7 acts in host protective processes and ROP1 is a rhoptry effector influencing the parasite load in vivo [20]. The data also suggest that LMO7 recruits ROP1 to its site of action and that ROP1 is likely to disrupt LMO7-dependent processes by blocking its interaction with natural partners. Although these results represent only a small piece of the puzzle, it can already be assumed that cryptosporidia hijack host actin similar to some enteropathogenic bacteria (*Salmonella* and *Escherichia coli*). This could explain why cryptosporidia exclusively prefer epithelial cells since these host cells possess a robust cortical skeleton, which allows the parasite to further stimulate actin polymerisation [20].

### 2.3. Modulation of Host Cell Apoptosis

However, manipulation of host cell actin polymerisation is not the only mechanism by which cryptosporidia affect the inner processes of the host cell to their own benefit. Modulation of host cell apoptosis is of particular interest and appears to be *Cryptosporidium*-stage dependent. First, the invading stages promote host cell apoptosis [39]. By inducing mild apoptosis at the expense of epithelial cell necrosis at the beginning of parasitisation, the parasite can reduce host inflammatory responses, thereby increasing its chances of survival and proliferation [40,41]. The subsequent trophozoite stages, on the other hand, inhibit apoptosis by producing anti-apoptotic factors. It occurs at a specific time of infection, when the parasites strictly depend on the host cell metabolites for their own growth and maturation [39]. The parasitisation leads to an upregulation of gene-encoding inhibitors of some apoptotic proteins and active suppression of the host cell apoptotic response. This is further supported by the fact that cryptosporidia prefer dividing cells (S/G2/M phase) to stationary cells for their initial asexual development and in addition, parasitisation of cell cultures by these parasites further induces cell division [42]. This could be due to their dependence on host cell metabolites, but also due to possible changes in cell surface proteins (mediating the zoite attachment) during the cell cycle. Lastly, when *Cryptosporidium* is ready to release infective stages, it promotes the host cell apoptosis again and uses this process to exit from the host cell [43]. Our own in vivo observations support this pro-apoptotic manipulation in both gastric and intestinal cryptosporidia. Interestingly, apoptosis is reactivated in both parasitised host cells and non-parasitised neighbouring cells during this later phase of infection [44,45,46] via a Fas/FasL-dependent mechanism likely involving both autocrine and paracrine pathways [46].

### 2.4. Immune Evasion

*Cryptosporidium* residency in a PS on the apical surface of epithelial cells is considered as one of the parasite immune evasion strategies [37]. The parasite is thus protected from the immune cells in the lamina propria that could facilitate parasite clearance [47]. Nevertheless, in an immunocompetent human host, *Cryptosporidium* is eventually cleared. As far as we know, the key molecule of the host immune defence responsible for this effect is IFNγ [47,48]. Its important source is CD4+ T lymphocytes, supported by the fact that cryptosporidiosis is more severe in AIDS patients [49]. However, other immune cells, such as NK cells and CD8+ T cells, contribute to its production as well, and, surprisingly, an important source are also macrophages stimulated by IL-18 produced by infected enterocytes [47,50]. Parasitised enterocytes respond to IFNγ; they can clear the attached parasites and secrete molecules that can prevent further parasite attachment and thus limit the parasite load [47,48]. The mechanism may lie in the depletion of intracellular iron as an essential nutrient for the parasite [48]. Enterocytes further assist in parasite clearance by expression of inducible nitric oxide synthase (iNOS) and subsequent production of nitric oxide (NO) as well as by the release of antimicrobial peptides (e.g., LL-37, beta-defensin-2) into the lumen of the gastrointestinal tract [49]. 

*Cryptosporidium* fights back by interfering with the IFNγ signalling pathway. In the infected enterocytes, the parasite depletes STAT-1 as the major component of IFNγ signalling, thus impairing the effect of IFNγ on the host cell [51]. Parasites are also able to modulate host gene transcription to transiently supress the expression of the antimicrobial peptide (beta-defensin-1) or IL-33. Host gene transcription is modulated by parasite RNA transcripts delivered to the host nucleus through heat shock protein 70-mediated nuclear importing mechanisms [1,50,52,53].

The epicellular development at the microvillous surface within a host-derived capsule appears to be a self-protective strategy of this apicomplexan parasite against the hostile conditions of host’s gastrointestinal environment [3,4,37]. It may also limit the exposure of parasite antigens [49], as observed in epicellular fish coccidia. In fish, resistance is acquired after primary exposure to the intestinal coccidian *Goussia carpelli*, residing in a deeper niche within the intestinal epithelium of common carp, due to a strong adaptive immune response preventing the reinfection (effective even under immunosuppression). Similar adaptive response, however, has not been demonstrated for epicellular coccidian *G. ameliae* from alewives fish, suggesting that it stimulates a less efficient adaptive immune response [32]. 

As a metaphor for this epicellular parasitism strategy in some apicomplexans, the phrase “dressed—undressed” according to the German fairy tale “The Peasant’s Wise Daughter” could be used. In this fairy tale, a clever girl solved the king’s riddle to visit him “neither naked nor dressed” by coming wrapped in a fishing net. It is also likely that this unique niche of *Cryptosporidium* endogenous stages is the main reason for the limited efficacy of tested chemotherapeutics, including anti-*Cryptosporidium* therapy with nitazoxanide, the only drug approved for human patients [54].

## 3. Chicken-and-Egg Dilemma: Protists on the Way in or out? 

Due to its superficial localisation on the host epithelium, *Cryptosporidium* can be considered a minimally invasive pathogen [5,55]. Some cryptosporidia parasitising the fish gastrointestinal tract, however, appear to have a greater invasive potential. Although their earlier stages develop epicellularly without apparent damage to host tissue, the sporulation takes place deeply within the host epithelium, rarely in the subepithelial connective tissue [55,56]. *C. scophthalmi* induces extensive epithelial destruction with intraepithelial sporogonial stages producing severe lesions in the parasitised intestine, accompanied by a strong inflammatory response. The host response to *Cryptosporidium* infection, however, may differ even in the same host, depending on parasite species and target host organ. Although heavy infections in fish result in massive epithelial necrosis and sloughing of epithelial cells, *C. molnari* preferring the stomach mucosa induces no inflammation and cellular rection. This process may be an adaptation needed for releasing the parasite transmission stages, since the focal epithelial necrosis and sloughing are associated with the release of oocysts into the gastrointestinal lumen. It has been observed also in other two epicellular coccidia, *Goussia ameliae* and *G. alosii*, parasitising the pyloric cecum and the posterior intestine of alewives. Despite the epicellular position of their earlier stages, oocysts of these eimeriid coccidia appeared slightly more embedded within the epithelium [32]. Similarly, meront and oocyst stages of certain intestinal cryptosporidia of guinea pigs and piglets have also been observed to be immersed deeply, presumably being intracytoplasmic [10,17].

Two explanations have been proposed for this phenomenon: (i) the intracytoplasmic localisation was secondary due to invagination of parasite into the host cell cytoplasm [17], and (i) endocytosis and transport of cryptosporidia by membranous epithelial cells (M cells) from lumen to the underlying lymphoid cells [10,57,58]. M cells, mainly found in the epithelium overlying mucosal lymphoid tissues, constitute the main cellular machinery for the selective sampling and transport of microbes and antigens for mucosal immunosurveillance [59]. However, due to the ability of M cells to effectively violate the mucosal barrier, certain pathogens may exploit them as an entry portal [60]. Of particular interest is that *Cryptosporidium* trophozoites and merozoites were identified within the cytoplasm of M cells, as partially digested parasites associated with subjacent macrophages [10] that accumulate during infection in the lamina propria. However, some of these engulfed parasites were found intact and possibly survived [61]. 

**Figure 3:**
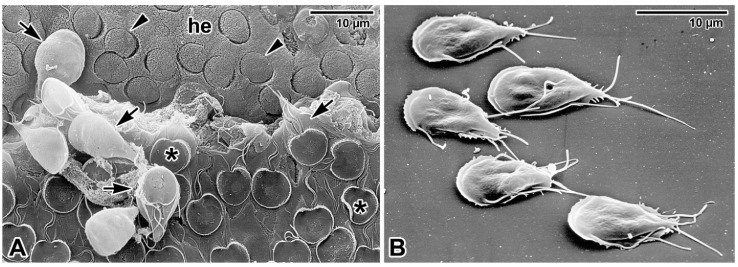
Adhesion of *Giardia intestinalis* trophozoites. (**A**) Trophozoites (black arrows) parasitising the intestinal epithelium of BALB/c mice. The microvillous surface of host epithelium (he) left after detached parasites shows numerous impressions (black arrowheads) in the shape of a ventral adhesive disk (asterisk). Scanning electron microscopy. (**B**) Trophozoites adhered to a microscopic slide. Scanning electron microscopy. Both electron micrographs courtesy of prof. Břetislav Koudela.

Similar invasive potential has been observed in another parasite with close epicellular relationship with enterocytes—*Giardia* (Diplomonadida). Although *Giardia* is generally referred to as extracellular parasite [62], its trophozoites cross the mucus layer protecting the epithelium surface and live attached on duodenal and jejunal epithelial cells using a ventral adhesive disk (Figure 3A), and should be thus more accurately referred to as epicellular parasites. 

It was first hypothesised that they adhere to epithelial microvilli to resist luminal flow while feeding on nutrients from intestinal fluid [62]. Nevertheless, *Giardia* trophozoites attach to enterocytes strongly via a suction-based mechanism involving the ventral disk and flagellar movements, but more importantly also via chemical bonds involving proteins and lipid raft membrane microdomains [62]. Moreover, the trophozoites can even disrupt epithelial tight junctions and induce caspase-3-dependent apoptosis of affected enterocytes; the situation is more pronounced in host-*Giardia* nonspecific combination or mixed infections by different *Giardia* genotypes [63]. Although *Giardia* trophozoites can adhere to any surface including glass or plastic (Figure 3B), the attachment to enterocytes is obligatory for the establishment of infection and the colonisation of the small intestine [62,64,65]. In addition, attached trophozoites are capable of the direct modification of enterocytes intracellular processes, and thus fully meet the epicellular localisation as defined in the introduction of this review.

Similar to cryptosporidia, *Giardia* usually does not invade the epithelial barrier, however in certain circumstances it may invade the mucosa and submucosa [62,66,67,68]. Deeper penetration of intestinal epithelium may be due to trophozoites accidentally entering epithelium disruptions/cavities left after desquamations [69]. Trophozoites may also take advantage of the epithelial discontinuity due to mucus discharge by goblet cells or even actively secrete substances that facilitate invasion [67]. Occasionally, in highly infected mice, *Giardia* has been found in other organs such as the liver, heart, and brain intercellularly inside the tissues [69]. In hosts additionally suprainfected with *Plasmodium berghei*, *Giardia* invaded extraintestinal tissues in up to 80% of mice, indicating its diminished resistance to invasion under these specific circumstances.

The “On the Way In or Out” dilemma comes from the assumption that certain types of intracellular protist may have arisen initially as forms attached to the cell surface [70], but the exact opposite may be true, or alternatively all these parasitism strategies may have arisen independently, as recently hypothesised in Apicomplexa [71,72]. The “On the Way In” hypothesis assumes that the evolution of apicomplexan parasitism evolved from ancestral myzocytotic predation (piercing the prey cell with a rostrum, containing specialised organelles similar to those used by apicomplexans for invasion, and sucking its content) to myzocytotic extracellular parasitism (demonstrated in archigregarines and blastogregarines, and probably occurring in cryptosporidia via the feeder organelle), accompanied by the origin of epicellular parasitism, and progressed into intracellular parasitism [5,30]. However, it is also possible that intracellular and epicellular parasitism in Apicomplexa emerged independently [71,72] and evolutionary selection has simply favoured epicellular niches for these apicomplexans. Similarly, the intercellular form of parasitism in other protists, as seen in *Giardia* trophozoites under specific conditions described above [62,66,67,68,69], may have evolved from extracellular or epicellular ancestors. On the contrary, support for the “On the Way Out” hypothesis can be found in the fish parasite, *Eimeria anguillae*, in which during intracellular merogony the PV is expelled into the apical region of the host cell [73].

Regardless of their origin, however, it can be said that epicellular parasitic protists are well adapted to secure tight contact with the host cell. To some extent, they tend to modify host cell morphology and/or metabolic pathways to access nutrients necessary for multiplication and/or prevent expulsion of their stages from luminal sites of hollow organs, but still have a less negative effect on host tissue and fitness than intracellular ones [74], and from this perspective epicellular parasitism is advantageous [5]. Gastrointestinal parasites with epicellular development, for example, usually cause only local damage to individual cells and are thus much gentler to their host, and overall epithelial damage is negligible and easily repaired due to its high regenerative capacity [5]. This balances the fitness of both the parasite and the host in favour of successful parasite replication.

In either way, some of them, such as the epicellular parasites discussed herein, have not lost the ability to invade the host tissue or cell more aggressively, but do not commonly use it because significant damage to the host tissue/organ may bring them less benefit than a gentler form of parasitism causing weaker host defence.

## 4. The Darkest Place Is under the Candle: Taming of the Immune Cells by *Leishmania*

The adaptation for intracellular parasitism differs between parasite species and is tightly connected with the type of host cell, as well as with invading and survival strategies. Some parasites have a broader spectrum of host cell types. Examples of such parasites are *Trypanosoma cruzi* (Kinetoplastida) and *Toxoplasma gondii* (Apicomplexa). Some parasites, on the other hand, adapted to an intracellular parasitic lifestyle inside the limited spectrum of host cells. Such specialised parasites are *Leishmania* spp. that harness the phagocytic cells of the immune system. There are several phagocytic cells in the immune system; two of them play a more important role in the *Leishmania* life cycle—neutrophils and monocytes/macrophages.

### 4.1. Early Survival in Neutrophils

Neutrophils play an important role in the hide-and-seek game in the early phase of infection. They are recruited to the transmission site within hours, earlier than other immune cells from the peripheral blood [75,76]. They are able to engulf promastigotes and provide them with a provisional shelter [77], hiding them from the extracellular hostile environment until the arrival of the monocytes, the *Leishmania* main host cells. *Leishmania* persists in early infected neutrophils as a nonreplicating promastigote, and it does not transform into an amastigote form [77,78]. 

Once inside, *Leishmania* has to solve how to escape from the neutrophil effector functions—digestion and production of reactive oxygen species (ROS). Inside neutrophils, parasites inhabit the phagosome but also nonlytic compartments [77,78]. To survive intracellularly, promastigotes silence the microbicide functions of their host cells. They are capable of inhibiting phagosome maturation, ROS production, and prolonging the neutrophil lifespan until arrival of monocytes at the transmission site two–three days later [77,79]. *Leishmania* appears to hijack the host cell apoptotic pathway in a fashion similar to *Cryptosporidium*; the aim is to inhibit host cell apoptosis at the early stages of parasitisation, while benefiting from apoptosis-like host cell damage for further spreading [80,81,82]. 

Parasite-derived molecules responsible for these effects are surface molecules such as lipophosphoglycan (LPG), glycosylinositolphospholipids (GILPs), and the metalloprotease leishmanolysin (GP63). *Leishmania* is also equipped with a battery of enzymes with antioxidant function, e.g., trypanothion synthetase, tryapanothion reductase, peroxiredoxins, and superoxide dismutase [79] that protect the parasite against ROS. A growing body of evidence suggests that engulfed promastigotes also harness the intracellular signalling pathways in their favour [83]. As an example of this manipulation, *L. major*-infected primary human neutrophils were found to upregulate the expression of complement receptors 1 and 3 and therefore increase the phagocytosis of apoptotic cells from the surrounding environment. This leads to the inhibition of neutrophil defence reactions such as the production of ROS and the pro-inflammatory cytokine TNF-α [83].

### 4.2. Silent Entry into Monocytes and Macrophages

The main *Leishmania* host cells are attracted to the transmission site from the peripheral blood. These are monocytes and monocyte-derived macrophages. They are attracted to the site of infection by chemokines produced by dermal resident cells, infected neutrophils, and later—during the adaptive phase—also in response to IFN-γ [84,85,86]. Although IFN-γ modulates the immune response towards the Th1/pro-inflammatory type that should promote host protection, the monocytes stay alternatively activated even in this pro-inflammatory microenvironment, and thus permissive to *Leishmania* [85,86].

**Figure 4:**
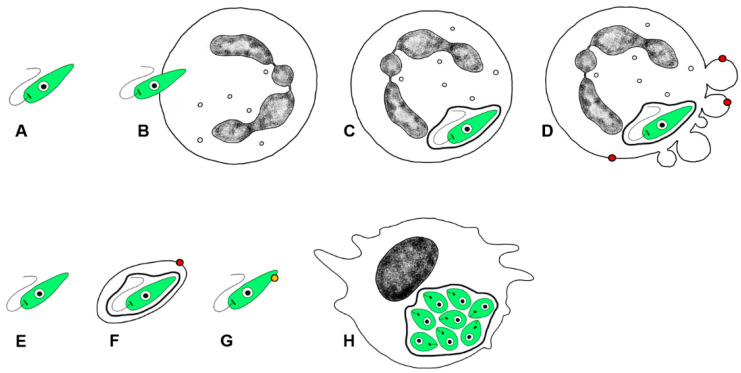
What is behind a silent entry? The infective inoculum, besides other factors, contains viable promastigotes represented as green cells (**A**). Neutrophils engulf promastigotes (**B**), which survive intracellularly in a parasitophorous vacuole represented by a thicker black line around the green parasite (**C**). Eventually, infected neutrophils become apoptotic, showing the phosphatidylserine on the outer side of the plasma membrane, represented as a red dot (**D**). *Leishmania* promastigotes can be phagocyted by their host cells, typically monocytes and macrophages, as free promastigotes (**E**), in neutrophil apoptotic bodies (**F**), or as apoptotic promastigotes expressing *Leishmania* phosphatidylserine analogue, represented as a yellow dot (**G**). Macrophages engulf *Leishmania* in the presence of apoptotic signals (either of neutrophil or *Leishmania* origin), which makes the microenvironment anti-inflammatory, thus supporting *Leishmania* survival and the establishment of infection (**H**).

Promastigotes apply a “silent entry” strategy to enter monocytes and macrophages (Figure 4). It is called a Trojan horse hypothesis, named after a trick used by a mythic Greek soldier, Odysseus, to conquer the town Troy after a ten-year-long siege. Odysseus hid himself inside a big wooden horse, which was offered as a gift for the Trojans, while the rest of the Odysseus army pretended to leave. Unaware of the danger, the Trojans took the wooden horse behind the town walls. During the night, Odysseus came out of the wooden horse and opened the gates for his soldiers and conquered the town. Analogically, a promastigote hidden inside an apoptotic neutrophil is engulfed by a macrophage without noticing the pathogen inside [75,87,88]. A Trojan rabbit hypothesis has been introduced as well [76,89], describing a situation where a promastigote escapes from the dying neutrophil and is engulfed by a macrophage as an extracellular parasite, but in the anti-inflammatory environment made by surrounding apoptotic signals, either of host or *Leishmania* origin [79]. 

In fact, *Leishmania* shows a form of altruistic interaction, ensuring the survival of infective stages—the metacyclic promastigotes. The infective inoculum contains a population of parasites that expose phosphatidylserine analogue as an “eat me” signal [90,91]. This apoptotic non-viable subpopulation of leishmania thus helps to silence the phagocyte pro-inflammatory and leishmanicide response, thereby allowing the establishment of infection from the viable non-apoptotic metacyclic promastigotes [92,93]. 

All three theories—Trojan horse, Trojan rabbit, and *Leishmania* altruism—explain why the infected macrophages stay “silent”. The process of efferocytosis (an engulfment of apoptotic cells/bodies) does not alarm the microbicide functions of macrophages. Thus, the macrophages neglect *Leishmania* residency and even support their multiplication.

### 4.3. Parasite Internalisation into Monocytes and Macrophages and Modulation of Host Cell Actin 

*Leishmania* intracellular parasitism in the vertebrate host seems to depend on the host cell’s ability to phagocytose. The entry into macrophages is based on the interactions between promastigote surface molecules and macrophage surface receptors and likely varies between *Leishmania* species [94]. *Leishmania* can even facilitate this process; it can make itself “tastier”. For this purpose, *Leishmania* harnesses opsonins such as complement, and during the later stage of infection also specific antibodies [95]. 

The process of internalisation is essential for shedding the major components of the promastigotes surface coat, LPG and GP63, and their spreading beyond the parasitophorous vacuole (PV) and redistribution in the host cell endoplasmic reticulum [96]. The metacyclic promastigotes and—in the later stage of infection also amastigotes—are internalised by an actin-dependent process, with macrophage actin most likely sequentially polymerising and depolymerising along parasite body during internalisation [97]. Actin filaments transiently accumulate around the parasites undergoing phagocytosis, forming a phagocytic cup rich in F-actin that usually rapidly disappears, exposing the phagosomal membrane to interact with early endosomes [98]. In wild-type *L. donovani*, however, parasite LPG is transferred to both the macrophage plasma membrane and the phagosomal membrane, delaying the PV maturation by interfering with disassembly of periphagosomal F-actin and inducing its polymerisation [99]. This creates a physical barrier for phagosome-endosome fusion and prevents vesicular trafficking to/from the phagosome [100]. While this LPG-dependent accumulation of periphagosomal F-actin correlates with an impaired recruitment of the lysosomal markers (LAMP1, PKCα) to the phagosome [100], other studies revealed that Cdc42 (a small GTPase of the Rho family involved in regulation of the cell cycle) is retained on phagosomes in the presence of LPG and is thus responsible for the accumulation of F-actin [101,102]. Moreover, LPG causes a disorganization of the phagosomal membrane [102]. Importantly, the F-actin accumulates around phagosomes harbouring wild-type *L. donovani* promastigotes but disassembles in those containing the LPG-defective mutant [100]. 

Actin turnover is modulated also by NO—the key leishmanicidal molecule of macrophages—which likely plays an important role in forcing the phagosome maturation by rupturing the periphagosomal actin. Hence, formation of F-actin-dependent blockade of phagosome maturation could be another mechanism that *Leishmania* uses to inhibit iNOS activity by LPG [99]. 

*Leishmania*-induced modulation of host cell F-actin dynamics results in impaired movement and directional migration of infected macrophages [103]. However, the use of host F-actin may vary in various *Leishmania* species because no accumulation of actin has been reported around phagosomes with *L. amazonensis* promastigotes [98]. 

Other cytoskeletal elements may also determine the further development of a battle between the infected cell and *Leishmania*. For example, in macrophages infected by *L. infantum*, vacuolar movement along host cell microtubules for the phagosome trafficking towards the endolysosomal pathway is required to mature into a tight-fitting PV crucial for parasite proliferation [104].

### 4.4. Formation of Parasitophorous Vacuole

At the end of the internalisation the formation of PV is initiated. The process precedes the transformation of *Leishmania* into an amastigote form and its subsequent multiplication. The *Leishmania* PV is derived partially from the host molecules of the endocytic and secretory pathways and shows some phagolysosomal properties [105,106]. PV acquires endoplasmic reticulum components by fusing with vesicles derived from the early secretory pathway. Disruption of this interaction leads to inhibition of PV development and also to reduced parasite replication in host cells. Hence, *Leishmania* PVs are hybrid acidic compartments, containing certain lysosomal enzymes, endoplasmic reticulum molecules, and are limited by a membrane enriched in late endosomal/lysosomal proteins of small GTP-binding proteins [106,107,108]. The initial steps during PV formation are, at least partially, regulated by the parasites; however, while the parasite-derived proteins were identified as distributed throughout the host cell, little is known about the parasite molecules secreted into the PV [105,109]. 

**Figure 5:**
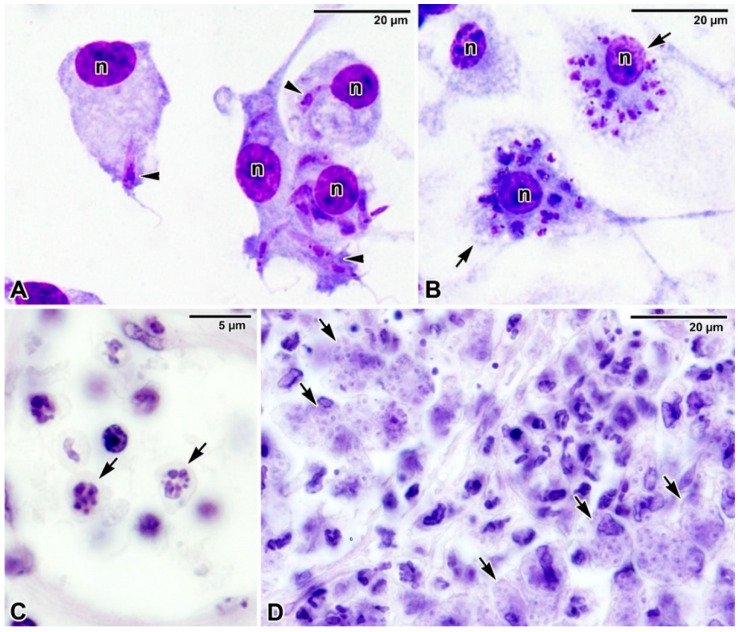
Intracellular localisation of *Leishmania*. (**A**,**B**) Giemsa-stained smear preparation showing (**A**) macrophages with invading *Leishmania donovani* promastigotes (arrowheads) and (**B**) three macrophages, two of which are heavily parasitised by *L. donovani* amastigotes (arrows). *n*—macrophage nucleus. Light microscopy. (**C**,**D**) Haematoxylin-eosin-stained histological sections of the BALB/c mice ear showing numerous macrophages parasitised by *Leishmania major* amastigotes (arrows). Light microscopy. Micrographs (**A**,**B**) courtesy of Dr. Tereza Leštinová.

Some of the PVs features are shared between species, some are *Leishmania* species-specific, varying morphologically and/or functionally [109]. The size of PVs varies among *Leishmania* species, with a very large PV harbouring multiple amastigotes (PV continuously enlarges as the parasite replicates, e.g., *L. mexicana* complex) and a small and tight-fitting PV having a single amastigote that splits after parasite replication (e.g., *L. major*, *L. donovani* complex) [108,110]. The PV size correlates with parasite survival, as large PV dilutes the nitric oxide (NO) intravacuolar concentration and thus reduces its concentration to a level tolerated by the parasite [110]. Once inside the host cell, *Leishmania* promastigotes (Figure 5A) undergo transformation into amastigotes (Figure 5B–D) that are better adapted to intracellular life and—in contrast to promastigotes—can even multiply in the vertebrate host. 

Intracellular trypanosomatid parasites require an acidic environment for intracellular development and recruit lysosome markers to the PV, thus remodelling and subverting the host endolysosomal pathway in their favour [111]. However, while PV in *T. cruzi* has a transient protective function as parasites are released to host cell cytosol where they transform into amastigotes and multiply, *Leishmania* PV secures the parasite for the whole intracellular residency, even during their escape from host cells and infection of new ones [111]. *Leishmania* amastigotes are acidophilic parasites with a metabolic optimum at the acidic pH which is constantly maintained inside the PV. They have several possible strategies to resist host proteases [109,111]. Moreover, *Leishmania* amastigotes themselves may be involved in acidifying the PV environment by the expression of a P-type H+ ATPase in their plasma membrane, which expels protons from the parasite’s cytosol into the PV interior [109]. 

### 4.5. Immune Evasion

To multiply inside the PV, *Leishmania* consume the host cell iron and arginine-derived polyamines as essential nutrients [112,113]. Both *Leishmania* and macrophage arginases contribute to arginine processing into polyamines [112,114], meanwhile inhibiting leishmanicide NO production by competing with macrophage NO synthase for arginine as a common substrate [112,115]. 

To stay hidden from the other immune cells that are in search of infected cells, *Leishmania* is able to impair macrophage’s antigen presentation process by disrupting it at different levels. The phagolysosomal nature of PV has properties similar to those known for MHC class II compartments that are capable of processing antigens and generating complexes of MHC class II and an antigenic protein for transport to the host cell surface and presentation to T cells [98,107]. Hence, the phagosome maturation is also essential to initiate the adaptive immune responses, and *Leishmania* parasites have evolved mechanisms to escape this potentially hazardous antigen presentation process. Amastigotes can sequester and degrade the antigens from the MHC II pathway, they can also downregulate the expression of MHC class II and of costimulatory molecules, and even inhibit migration of macrophages into the draining lymph nodes where the interaction with T lymphocytes during antigen presentation process takes place [79,86,112]. 

Infected monocytes are further employed to promote *Leishmania* infection; they are forced to keep the alternatively activated status (e.g., express more IL-4R), survive longer, and support subsequent monocyte recruitment via chemokines production [82,86,112]. The parasite can also downregulate the classical macrophage autophagy and at the same time use an alternative mechanistic target of rapamycin (mTOR)-independent pathway to induce autophagy with the possibility of finetuning its timing and intensity according to its own needs [81]. *Leishmania* is capable of modulating the macrophage’s signalling pathways and transcription factors to accomplish all these effects [112,116]. 

The surrounding environment is further modulated in favour of the replicating parasite by extracellular vesicles emerging from the infected cell. These vesicles carry the immunosuppressive cargo with anti-inflammatory effect; however, their exact effect depends on the particular *Leishmania* species [84,117]. Phagocytosis of these exosomes by uninfected macrophages can prevent their activation [94]. Through the parasite virulence factors transported by exosomes, *Leishmania* parasites that reside within PV can up or downregulate some of the uninfected macrophage signalling pathways, and subsequently innate and adaptive immune responses [118].

### 4.6. Apoptosis as a Dissemination Strategy

Amastigotes spreading takes place when the host macrophage delivers warning signs of imminent apoptosis. The parasites leave the macrophage within host-derived membrane blebs rich in phagolysosomal membrane components. After leaving host apoptotic macrophage, *L. amazonensis* remains associated with the host lysosomal components on its surface that trigger anti-inflammatory cytokine production by surrounding non-apoptotic macrophages [80,111]. The amastigotes are thus taken up by surrounding macrophages in this anti-inflammatory milieu, again elegantly subverting host defence [80].

Apoptotic mimicry is another evasion strategy of *Leishmania* amastigotes to ensure the overall survival of the parasite population in the host body. Amastigotes of *L. amazonensis* expose an apoptotic signal —phosphatidylserine—while retaining viability and even infectivity [119,120]. These parasites are recognised as apoptotic, leading to induction of an anti-inflammatory macrophage response. Phosphatidylserine is efficient in regulating inflammation and promoting immune tolerance, which is beneficial for infection establishment and parasite dissemination [92].

### 4.7. Other Cells That Can Host Leishmania

Although *Leishmania* is such a specialised intracellular parasite, it has also been observed inside other cell types [75,121]. The first line of defence against microbes invading the skin is provided by dermal tissue resident immune cells—macrophages and mast cells. These cells are able to produce and release immunomodulatory molecules that, for example, support subsequent neutrophil recruitment to the infection site. During the first hours of *Leishmania* infection, dermal tissue resident macrophages play a similar role as neutrophils; they provide a provisional shelter for promastigotes, but parasites do not replicate in this niche [75]. Mast cells are also able to engulf *Leishmania* promastigotes and even support their multiplication [122]; however, their exact role in leishmaniasis depends on infecting *Leishmania* species as well as on the host genetic background [123,124]. 

Recently, a dual role of neutrophils in *Leishmania* infection was introduced; during the chronic phase of infection, even neutrophils can support *Leishmania* replication. This has been observed for *L. amazonensis*; neutrophils were able to engulf the amastigotes, which survived intracellularly and even multiplied there [125,126]. 

Although dendritic cells and eosinophils can also phagocyte leishmania, the research suggests they have no role in parasite multiplication nor in *Leishmania* survival but rather in shaping the local inflammatory response as well as the onset of anti-*Leishmania* adaptive immunity [112,124]. 

Last but not least, *Leishmania* has been observed also in nonprofessional phagocytic cells, such as fibroblasts [121]. Although *Leishmania* intracellular parasitism seems to be dependent on the host cell’s ability to phagocyte, some studies indicate that promastigotes may actively participate in the phagocytic uptake by macrophage via their flagellar motility. The phagocytosis of *Leishmania* promastigotes appears to be polarised and induced by the interaction between the tip of the parasite’s flagellum and the target cell, resulting in formation of a pseudopod that initiates at the parasite flagellar tip and extends toward its cell body [127]. Persistent flagellar activity leads to a parasite intracellular reorientation, with its flagellum towards the macrophage periphery. Oscillations of the parasite could be the source of local plasma membrane injury observed at the parasite oscillation site, leading to a recruitment and exocytosis of host cell lysosomes involved in plasma membrane repair to maintain its integrity [127]. 

Using this mechanism of entry, *Leishmania* can also possibly enter fibroblasts. By wounding the fibroblasts´ plasma membrane, *L. amazonensis* promastigotes are unintentionally internalised in the follow-up process of membrane reparation [128]. Because fibroblasts have a long lifespan and they also have a limited ability to destroy the parasite, they may thus contribute to the persistence of infection [121]. The invasion of non-phagocytic cells (i.e., by mechanisms distinct from phagocytosis) immediately after inoculation can provide the parasite with a safe shelter to avoid the host innate immunity or to serve as a reservoir [128]. Thus, it is possible that other cell types also function as Trojan horses, especially in the early stages of *Leishmania* infection.

## 5. Conclusions

The parasite–host interactions are very dynamic as they reflect the balance between the host defence against the parasite and the rapid development and adaptation of the parasite to newly established conditions. The most successful parasites are those that can use their host without killing it. In this review, we discussed the adaptations to epicellular and intracellular parasitism with the example of two medically important parasites—*Cryptosporidium* and *Leishmania*—compared with adaptations described in other protists parasites. 

At this point, we also emphasise that it is crucial to consider the extra–, epi–, or intracellularity of parasites in the context of their complex life cycle and/or development in vivo under challenging natural conditions and in connection with their pathogenicity to host organisms, and not based solely on their ability to grow in artificial media during in vitro research [129], as is often applied to cryptosporidia. It has been postulated that the ability of pathogens to replicate in vitro requires metabolic pathways that differ from those involved in the in vivo development within the host organisms [129]. Similarly, *Leishmania* amastigotes from the axenic culture possess different metabolic characteristics than those isolated from the infected animal [130,131]. 

In addition, some stages in the life cycle of generally epicellular or extracellular pathogens may have invasive abilities, as shown in some *Cryptosporidium* and *Goussia* species. Under specific circumstances, even *Giardia* can be found intercellularly [62,66,67,68,69]. The ability to attack and multiply in different host cells and/or host environments (often manifested by parasites with dual life cycle comprising both the extracellular and intracellular phases) makes some of the infections much more severe [129] and may be among reasons for the reduced effectiveness of treatment.

In comparison to *Cryptosporidium*, *Leishmania* parasites use different survival strategies (Table 1) but to the very same goal—to successfully multiply in the host without killing it, thus infecting other hosts and continuing the life cycle. Regardless of the parasite localisation, these two parasites are both able to hijack the host cell actin (de)polymerisation and reorganisation as well as the host cell metabolic pathways to access nutrients necessary for the multiplication, and to tame the host cell defence mechanisms (e.g., apoptosis) in their favour. They can even hijack the signalling pathways to evade host immune response.

However, there are still many unanswered questions that need to be addressed to fully understand the sophisticated strategy of these parasites and, possibly, to employ them in disease control. Here, we emphasise some of them:

(i) What is the real invasive potential of epicellular parasites and what drives them to invade deeper tissues of some hosts (parasite virulence differing among species, host fitness/immunity status, or other non-considered factors)?
(ii) What is the reason for the limited efficacy of tested chemotherapeutics if cryptosporidiosis is an acute self-limiting infection in immunocompetent hosts?
(iii) What determines whether *Leishmania* promastigotes transform into the amastigote stage? Why can it not transform within dermal tissue-resident macrophages or neutrophils?
(iv) What is the exact composition of parasitophorous envelopes (parasitophorous sac or parasitophorous vacuole) in *Cryptosporidium*, *Leishmania*, and, consequently, in other parasites with similar localisation? How does the parasite contribute to its composition?

The detailed characterisation of specific adaptations may help in the future to design more effective prophylactic and treatment strategies for humans, as well as animal health management of cryptosporidiosis and leishmaniasis. 

## Figures and Tables

**Table 1:** Summary of the main features of the *Cryptosporidium* and *Leishmania* parasitism strategy discussed in this review.

Adaptation/Evasion	*Cryptosporidium*	*Leishmania*
Preferred cells for parasite development/multiplication	Microvillous surface of the epithelial cells (mostly gastrointestinal tract)	Monocytes/macrophages
Parasite localization in respect to the host cell	Epicellular (Above plasma membrane)	Intracellular (Below plasma membrane)
Parasite compartment	Parasitophorous sac	Parasitophorous vacuole
Parasite invasive apparatus	present	absent
Modulation of host signalling pathways	yes	yes
Modulation of host cell actin	yes	yes
Modulation of host cell apoptosis	yes	yes

## Data Availability

No new data were created or analysed in this study. Data sharing is not applicable to this article.

## Abbreviations

H+ ATPase	Hydrogen-exporting Adenosine Triphosphate Phosphohydrolase
GIPLs	Glycosylinositolphospholipids
GP63	Glycoprotein 63, syn. *Leishmania* metalloproteinase, leishmanolysin
IFN-γ	Interferon gamma
IL	Interleukin
iNOS	Inducible Nitric Oxide Synthase
LAMP	Lysosome-Associated Membrane glycoproteins
LMO7	LIM domain Only 7
LPG	Lipophosphoglycan
NK	Natural Killer cells
NO	Nitric Oxide
PKC	Protein Kinase C
PS	Parasitophorous Sac
PV	Parasitophorous Vacuole
RON	Rhoptry Neck Protein
ROP	Rhoptry (bulb) Protein
ROS	Reactive Oxygen Species
STAT-1	Signal Transducer and Activator of Transcription 1
TNF-α	Tumor Necrosis Factor alpha, syn. cachexin

## References

[1] Ratcliffe M.J.H. (2016). Encyclopedia of Immunobiology.

[2] Briceño A.L., Contreras Z.P., Vera D.D., Briceño R.M., Pru E.P., Ceccherini-Nelli L., Matteoli B. (2012). Tissue culture to assess bacterial enteropathogenicity. Biomedical Tissue Culture.

[3] Valigurová A., Jirků M., Koudela B., Gelnar M., Modrý D., Šlapeta J. (2008). Cryptosporidia: Epicellular parasites embraced by the host cell membrane. Int. J. Parasitol..

[4] Valigurová A., Hofmannová L., Koudela B., Vávra J. (2007). An ultrastructural comparison of the attachment sites between *Gregarina steini* and *Cryptosporidium muris*. J. Eukaryot. Microbiol..

[5] Valigurová A., Paskerova G.G., Diakin A., Kováčiková M., Simdyanov T.G. (2015). Protococcidian *Eleutheroschizon duboscqi*, an unusual apicomplexan interconnecting gregarines and cryptosporidia. PLoS ONE.

[6] Dubremetz J.F., Garcia-Reguet N., Conseil V., Fourmaux M.N. (1998). Apical organelles and host-cell invasion by Apicomplexa. Int. J. Parasitol..

[7] Bargieri D., Lagal V., Andenmatten N., Tardieux I., Meissner M., Ménard R. (2014). Host cell invasion by apicomplexan parasites: The junction conundrum. PLoS Pathog..

[8] Umemiya R., Fukuda M., Fujisaki K., Matsui T. (2005). Electron microscopic observation of the invasion process of *Cryptosporidium parvum* in severe combined immunodeficiency mice. J. Parasitol..

[9] Borowski H., Thompson R.C., Armstrong T., Clode P.L. (2010). Morphological characterization of *Cryptosporidium parvum* life-cycle stages in an in vitro model system. Parasitology.

[10] Marcial M.A., Madara J.L. (1986). *Cryptosporidium*: Cellular localization, structural analysis of absorptive cell-parasite membrane-membrane interactions in guinea pigs, and suggestion of protozoan transport by M cells. Gastroenterology.

[11] Barta J.R., Thompson R.C.A. (2006). What is *Cryptosporidium*? Reappraising its biology and phylogenetic affinities. Trends Parasitol..

[12] Carreno R.A., Martin D.S., Barta J.R. (1999). *Cryptosporidium* is more closely related to the gregarines than to coccidia as shown by phylogenetic analysis of apicomplexan parasites inferred using small-subunit ribosomal RNA gene sequences. Parasitol. Res..

[13] Clode P.L., Koh W.H., Thompson R.C.A. (2015). Life without a host cell: What is *Cryptosporidium*?. Trends Parasitol..

[14] Melicherová J., Hofmannová L., Valigurová A. (2018). Response of cell lines to actual and simulated inoculation with *Cryptosporidium proliferans*. Eur. J. Protistol..

[15] Ryan U., Paparini A., Monis P., Hijjawi N. (2016). It’s official-*Cryptosporidium* is a gregarine: What are the implications for the water industry?. Water Res..

[16] Lumb R., Smith K., Odonoghue P.J., Lanser J.A. (1988). Ultrastructure of the attachment of *Cryptosporidium* sporozoites to tissue-culture cells. Parasitol. Res..

[17] Koudela B., Vitovec J., Sterba J., Milacek P. (1989). An unusual localization of developmental stages of *Cryptosporidium parvum* Tyzzer, 1912 in the cells of small intestine of a gnotobiotic piglet. Folia Parasitol..

[18] Valentini E., Cherchi S., Possenti A., Dubremetz J.F., Pozio E., Spano F. (2012). Molecular characterisation of a *Cryptosporidium parvum* rhoptry protein candidate related to the rhoptry neck proteins TgRON1 of *Toxoplasma gondii* and PfASP of *Plasmodium falciparum*. Mol. Biochem. Parasitol..

[19] Singh P., Mirdha B.R., Srinivasan A., Rukmangadachar L.A., Singh S., Sharma P., Gururao H., Luthra K. (2015). Identification of invasion proteins of *Cryptosporidium parvum*. World J. Microbiol. Biotechnol..

[20] Guérin A., Roy N.H., Kugler E.M., Berry L., Burkhardt J.K., Shin J.-B., Striepen B. (2021). *Cryptosporidium* rhoptry effector protein ROP1 injected during invasion targets the host cytoskeletal modulator LMO7. Cell Host Microbe.

[21] Beyer T.V., Svezhova N.V., Sidorenko N.V., Khokhlov S.E. (2000). *Cryptosporidium parvum* (Coccidia, Apicomplexa): Some new ultrastructural observations on its endogenous development. Eur. J. Protistol..

[22] Forney J.R., DeWald D.B., Yang S.G., Speer C.A., Healey M.C. (1999). A role for host phosphoinositide 3-kinase and cytoskeletal remodeling during *Cryptosporidium parvum* infection. Infect. Immun..

[23] Landsberg J.H., Paperna I. (1986). Ultrastructural study of the coccidian *Cryptosporidium* sp. from stomachs of juvenile cichlid fish. Dis. Aquat. Organ..

[24] O’Hara S.P., Small A.J., Chen X.M., LaRusso N.F. (2008). Host cell actin remodeling in response to *Cryptosporidium*. Subcell. Biochem..

[25] Perkins M.E., Riojas Y.A., Wu T.W., Le Blancq S.M. (1999). CpABC, a *Cryptosporidium parvum* ATP-binding cassette protein at the host-parasite boundary in intracellular stages. Proc. Natl. Acad. Sci. USA.

[26] Tzipori S., Griffiths J.K. (1998). Natural history and biology of *Cryptosporidium parvum*. Adv. Parasitol..

[27] Dyková I., Lom J. (1981). Fish coccidia: Critical notes on life cycles, classification and pathogenicity. J. Fish. Dis..

[28] Lukes J. (1992). Life cycle of *Goussia pannonica* (Molnar, 1989) (Apicomplexa, Eimeriorina), an extracytoplasmic coccidium from the white bream Blicca bjoerkna. J. Protozool..

[29] Molnar K., Baska F. (1986). Light and electron microscopic studies on *Epieimeria anguillae* (Léger & Hollande, 1922), a coccidium parasitizing the European eel, *Anguilla anguilla* L.. J. Fish Dis..

[30] Valigurová A., Florent I. (2021). Nutrient acquisition and attachment strategies in basal lineages: A tough nut to crack in the evolutionary puzzle of Apicomplexa. Microorganisms.

[31] Bartošová-Sojková P., Oppenheim R.D., Soldati-Favre D., Lukeš J. (2015). Epicellular apicomplexans: Parasites “on the way in”. PLoS Pathog..

[32] Lovy J., Friend S.E. (2015). Intestinal coccidiosis of anadromous and landlocked alewives, *Alosa pseudoharengus*, caused by *Goussia ameliae* n. sp. and *G. alosii* n. sp. (Apicomplexa: Eimeriidae). Int. J. Parasitol. Parasites Wildl..

[33] Carruthers V.B., Tomley F.M. (2008). Microneme proteins in apicomplexans. Subcell. Biochem..

[34] Ben Chaabene R., Lentini G., Soldati-Favre D. (2021). Biogenesis and discharge of the rhoptries: Key organelles for entry and hijack of host cells by the Apicomplexa. Mol. Microbiol..

[35] Dogga S.K., Bartošová-Sojková P., Lukeš J., Soldati-Favre D. (2015). Phylogeny, Morphology, and metabolic and invasive capabilities of epicellular fish coccidium *Goussia janae*. Protist.

[36] Bonnin A., Dubremetz J.F., Camerlynck P. (1991). Characterization of microneme antigens of *Cryptosporidium parvum* (Protozoa, Apicomplexa). Infect. Immun..

[37] Melicherová J., Ilgová J., Kváč M., Sak B., Koudela B., Valigurová A. (2014). Life cycle of *Cryptosporidium muris* in two rodents with different responses to parasitization. Parasitology.

[38] Huang B.Q., Chen X.M., LaRusso N.F. (2004). *Cryptosporidium parvum* attachment to and internalization by human biliary epithelia in vitro: A morphologic study. J. Parasitol..

[39] Mele R., Morales M.A.G., Tosini F., Pozio E. (2004). *Cryptosporidium parvum* at different developmental stages modulates host cell apoptosis in vitro. Infect. Immun..

[40] McCole D.F., Eckmann L., Laurent F., Kagnoff M.F. (2000). Intestinal epithelial cell apoptosis following *Cryptosporidium parvum* infection. Infect. Immun..

[41] Sasahara T., Maruyama H., Aoki M., Kikuno R., Sekiguchi T., Takahashi A., Satoh Y., Kitasato H., Takayama Y., Inoue M. (2003). Apoptosis of intestinal crypt epithelium after *Cryptosporidium parvum* infection. J. Infect. Chemother..

[42] Widmer G., Yang Y.L., Bonilla R., Tanriverdi S., Ciociola K.M. (2006). Preferential infection of dividing cells by *Cryptosporidium parvum*. Parasitology.

[43] Ojcius D.M., Perfettini J.L., Bonnin A., Laurent F. (1999). Caspase-dependent apoptosis during infection with *Cryptosporidium parvum*. Microbes Infect..

[44] Chen X.M., Levine S.A., Tietz P., Krueger E., McNiven M.A., Jefferson D.M., Mahle M., LaRusso N.F. (1998). *Cryptosporidium parvum* is cytopathic for cultured human biliary epithelia via an apoptotic mechanism. Hepatology.

[45] Widmer G., Corey E.A., Stein B., Griffiths J.K., Tzipori S. (2000). Host cell apoptosis impairs *Cryptosporidium parvum* development in vitro. J. Parasitol..

[46] Chen X.M., Gores G.J., Paya C.V., LaRusso N.F. (1999). *Cryptosporidium parvum* induces apoptosis in biliary epithelia by a Fas/Fas ligand-dependent mechanism. Am. J. Physiol..

[47] Crawford C.K., Kol A. (2021). The mucosal innate immune response to *Cryptosporidium parvum*, a global one health issue. Front. Cell. Infect. Microbiol..

[48] Petry F., Jakobi V., Tessema T.S. (2010). Host immune response to *Cryptosporidium parvum* infection. Exp. Parasitol..

[49] Quach J., Chadee K., Mead J.R., Singer S.M., Ratcliffe M.J.H. (2016). Immunity to intestinal protozoa: *Entamoeba, Cryptosporidium*, and *Giardia*. Encyclopedia of Immunobiology.

[50] Barakat F.M., McDonald V., Di Santo J.P., Korbel D.S. (2009). Roles for NK cells and an NK cell-independent source of intestinal gamma interferon for innate immunity to *Cryptosporidium parvum* infection. Infect. Immun..

[51] Choudhry N., Korbel D.S., Edwards L.A., Bajaj-Elliott M., McDonald V. (2009). Dysregulation of interferon-γ-mediated signalling pathway in intestinal epithelial cells by *Cryptosporidium parvum* infection. Cell. Microbiol..

[52] Zaalouk T.K., Bajaj-Elliott M., George J.T., McDonald V. (2004). Differential regulation of defensin gene expression during *Cryptosporidium parvum* infection. Infect. Immun..

[53] Wang Y., Gong A.-Y., Ma S., Chen X., Li Y., Su C.-J., Norall D., Chen J., Strauss-Soukup J.K., Chen X.-M. (2016). Delivery of parasite RNA transcripts into infected epithelial cells during *Cryptosporidium* infection and its potential impact on host gene transcription. J. Infect. Dis..

[54] Ashigbie P.G., Shepherd S., Steiner K.L., Amadi B., Aziz N., Manjunatha U.H., Spector J.M., Diagana T.T., Kelly P. (2021). Use-case scenarios for an anti-*Cryptosporidium* therapeutic. PLoS Negl. Trop. Dis..

[55] Alvarez-Pellitero P., Quiroga M.I., Sitjà-Bobadilla A., Redondo M.J., Palenzuela O., Padrós F., Vázquez S., Nieto J.M. (2004). *Cryptosporidium scophthalmi* n. sp. (Apicomplexa: Cryptosporidiidae) from cultured turbot *Scophthalmus maximus*. Light and electron microscope description and histopathological study. Dis. Aquat. Organ..

[56] Alvarez-Pellitero P., Sitja-Bobadilla A. (2002). *Cryptosporidium molnari* n. sp. (Apicomplexa: Cryptosporidiidae) infecting two marine fish species, *Sparus aurata* L. and *Dicentrarchus labrax* L.. Int. J. Parasitol..

[57] Liebler E.M., Pohlenz J.F., Woodmansee D.B. (1986). Experimental intrauterine infection of adult BALB/c mice with *Cryptosporidium* sp.. Infect. Immun..

[58] Kennedy G.A., Kreitner G.L., Strafuss A.C. (1977). Cryptosporidiosis in three pigs. J. Am. Vet. Med. Assoc..

[59] Dillon A., Lo D.D. (2019). M Cells: Intelligent engineering of mucosal immune surveillance. Front. Immunol..

[60] Wang M., Gao Z., Zhang Z., Pan L., Zhang Y. (2014). Roles of M cells in infection and mucosal vaccines. Hum. Vaccin Immunother..

[61] De Sablet T., Potiron L., Marquis M., Bussière F.I., Lacroix-Lamandé S., Laurent F. (2016). *Cryptosporidium parvum* increases intestinal permeability through interaction with epithelial cells and IL-1β and TNFα released by inflammatory monocytes. Cell. Microbiol..

[62] Allain T., Amat C.B., Motta J.-P., Manko A., Buret A.G. (2017). Interactions of *Giardia* sp. with the intestinal barrier: Epithelium, mucus, and microbiota. Tissue Barriers.

[63] Koh W.H., Geurden T., Paget T., O’Handley R., Steuart R.F., Thompson R.C., Buret A.G. (2013). *Giardia duodenalis* assemblage-specific induction of apoptosis and tight junction disruption in human intestinal epithelial cells: Effects of mixed infections. J. Parasitol..

[64] Hernández-Sánchez J., Liñan R.F., del Rosario Salinas-Tobón M., Ortega-Pierres G. (2008). *Giardia duodenalis*: Adhesion-deficient clones have reduced ability to establish infection in Mongolian gerbils. Exp. Parasitol..

[65] Sousa M.C., Gonçalves C.A., Bairos V.A., Poiares-Da-Silva J. (2001). Adherence of *Giardia lamblia* trophozoites to Int-407 human intestinal cells. Clin. Diagn. Lab. Immunol..

[66] Brandborg L.L., Tankersley C.B., Gottieb S., Barancik M., Sartor V.E. (1967). Histological demonstration of mucosal invasion by *Giardia lamblia* in man. Gastroenterology.

[67] Reynoso-Robles R., Ponce-Macotela M., Rosas-López L.E., Ramos-Morales A., Martínez–Gordillo M.N., González-Maciel A. (2015). The invasive potential of *Giardia intestinalis* in an in vivo model. Sci. Rep..

[68] Saha T.K., Ghosh T.K. (1977). Invasion of small intestinal mucosa by *Giardia lamblia* in man. Gastroenterology.

[69] Owen R.L., Nemanic P.C., Stevens D.P. (1979). Ultrastructural observations on giardiasis in a murine model. I. Intestinal distribution, attachment, and relationship to the immune system of *Giardia muris*. Gastroenterology.

[70] Bannister L.H. (1979). The interactions of intracellular Protista and their host cells, with special reference to heterotrophic organisms. Proc. R. Soc. Lond. Ser. B Biol. Sci..

[71] Mathur V., Kolísko M., Hehenberger E., Irwin N.A.T., Leander B.S., Kristmundsson A., Freeman M.A., Keeling P.J. (2019). Multiple independent origins of apicomplexan-like parasites. Curr. Biol..

[72] Janouskovec J., Paskerova G.G., Miroliubova T.S., Mikhailov K.V., Birley T., Aleoshin V.V., Simdyanov T.G. (2019). Apicomplexan-like parasites are polyphyletic and widely but selectively dependent on cryptic plastid organelles. eLife.

[73] Benajiba M.H., Marques A., Lom J., Bouix G. (1994). Ultrastructure and sporogony of *Eimeria* (syn. *Epieimeria*) *anguillae* (Apicomplexa) in the Eel (*Anguilla anguilla*). J. Eukaryot. Microbiol..

[74] Eli A., Briyai O.F., Abowei J.F.N. (2012). A review of some parasite diseases of African fish gut lumen Protozoa, coccidioses, *Cryptosporidium* infections, Haemoprotozoa, Haemosporidia. Res. J. Appl. Sci. Eng. Technol..

[75] Chaves M.M., Lee S.H., Kamenyeva O., Ghosh K., Peters N.C., Sacks D. (2020). The role of dermis resident macrophages and their interaction with neutrophils in the early establishment of *Leishmania major* infection transmitted by sand fly bite. PLoS Pathog..

[76] Peters N.C., Egen J.G., Secundino N., Debrabant A., Kimblin N., Kamhawi S., Lawyer P., Fay M.P., Germain R.N., Sacks D. (2008). In vivo imaging reveals an essential role for neutrophils in leishmaniasis transmitted by sand flies. Science.

[77] Regli I.B., Passelli K., Hurrell B.P., Tacchini-Cottier F. (2017). Survival Mechanisms Used by Some *Leishmania* Species to Escape Neutrophil Killing. Front. Immunol..

[78] Gueirard P., Laplante A., Rondeau C., Milon G., Desjardins M. (2008). Trafficking of *Leishmania donovani* promastigotes in non-lytic compartments in neutrophils enables the subsequent transfer of parasites to macrophages. Cell. Microbiol..

[79] Cecílio P., Pérez-Cabezas B., Santarém N., Maciel J., Rodrigues V., Cordeiro da Silva A. (2014). Deception and manipulation: The arms of *Leishmania*, a successful parasite. Front. Immunol..

[80] Real F., Florentino P.T., Reis L.C., Ramos-Sanchez E.M., Veras P.S., Goto H., Mortara R.A. (2014). Cell-to-cell transfer of *Leishmania amazonensis* amastigotes is mediated by immunomodulatory LAMP-rich parasitophorous extrusions. Cell. Microbiol..

[81] Thomas S.A., Nandan D., Kass J., Reiner N.E. (2018). Countervailing, time-dependent effects on host autophagy promote intracellular survival of *Leishmania*. J. Biol. Chem..

[82] Getti G.T., Cheke R.A., Humber D.P. (2008). Induction of apoptosis in host cells: A survival mechanism for *Leishmania* parasites?. Parasitology.

[83] Salei N., Hellberg L., Köhl J., Laskay T. (2017). Enhanced survival of *Leishmania major* in neutrophil granulocytes in the presence of apoptotic cells. PLoS ONE.

[84] Pacheco-Fernandez T., Volpedo G., Verma C., Satoskar A.R. (2021). Understanding the immune responses involved in mediating protection or immunopathology during leishmaniasis. Biochem. Soc. Trans..

[85] Sacks D.L. (2020). The null hypothesis of IFN-γ and monocyte function in leishmaniasis. Cell Host Microbe.

[86] Carneiro M.B., Lopes M.E., Hohman L.S., Romano A., David B.A., Kratofil R., Kubes P., Workentine M.L., Campos A.C., Vieira L.Q. (2020). Th1-Th2 cross-regulation controls early *Leishmania* infection in the skin by modulating the size of the permissive monocytic host cell reservoir. Cell Host Microbe.

[87] Laskay T., van Zandbergen G., Solbach W. (2008). Neutrophil granulocytes as host cells and transport vehicles for intracellular pathogens: Apoptosis as infection-promoting factor. Immunobiology.

[88] van Zandbergen G., Klinger M., Mueller A., Dannenberg S., Gebert A., Solbach W., Laskay T. (2004). Cutting Edge: Neutrophil granulocyte serves as a vector for *Leishmania* entry into macrophages. J. Immunol..

[89] Ritter U., Frischknecht F., van Zandbergen G. (2009). Are neutrophils important host cells for *Leishmania* parasites?. Trends Parasitol..

[90] Wanderley J.L.M., Pinto da Silva L.H., Deolindo P., Soong L., Borges V.M., Prates D.B., de Souza A.P.A., Barral A., Balanco J.M.d.F., do Nascimento M.T.C. (2009). Cooperation between apoptotic and viable metacyclics enhances the pathogenesis of Leishmaniasis. PLoS ONE.

[91] Van Zandbergen G., Bollinger A., Wenzel A., Kamhawi S., Voll R., Klinger M., Müller A., Hölscher C., Herrmann M., Sacks D. (2006). *Leishmania* disease development depends on the presence of apoptotic promastigotes in the virulent inoculum. Proc. Natl. Acad. Sci. USA.

[92] El-Hani C., Borges V., Wanderley J.L., Barcinski M. (2012). Apoptosis and apoptotic mimicry in *Leishmania*: An evolutionary perspective. Front. Cell. Infect. Microbiol..

[93] Wanderley J.L.M., DaMatta R.A., Barcinski M.A. (2020). Apoptotic mimicry as a strategy for the establishment of parasitic infections: Parasite- and host-derived phosphatidylserine as key molecule. Cell Commun. Signal..

[94] Terrazas C., Oghumu S., Jha B.K., Natarajan G., Drew M., Denkers E.Y., Satoskar A.R., McGwire B.S., Ratcliffe M.J.H. (2016). Subverting immunity from the inside: Strategies of intracellular survival–protozoans. Encyclopedia of Immunobiology.

[95] Ueno N., Wilson M.E. (2012). Receptor-mediated phagocytosis of *Leishmania*: Implications for intracellular survival. Trends Parasitol..

[96] Carneiro M.B., Peters N.C. (2021). The paradox of a phagosomal lifestyle: How innate host cell-*Leishmania amazonensis* interactions lead to a progressive chronic disease. Front. Immunol..

[97] Courret N., Fréhel C., Gouhier N., Pouchelet M., Prina E., Roux P., Antoine J.C. (2002). Biogenesis of *Leishmania*-harbouring parasitophorous vacuoles following phagocytosis of the metacyclic promastigote or amastigote stages of the parasites. J. Cell Sci..

[98] Lerm M., Holm Å., Seiron Å., Särndahl E., Magnusson K.-E., Rasmusson B. (2006). *Leishmania donovani* requires functional Cdc42 and Rac1 to prevent phagosomal maturation. Infect. Immun..

[99] Winberg M.E., Rasmusson B., Sundqvist T. (2007). *Leishmania donovani*: Inhibition of phagosomal maturation is rescued by nitric oxide in macrophages. Exp. Parasitol..

[100] Holm Å., Tejle K., Magnusson K.-E., Descoteaux A., Rasmusson B. (2001). *Leishmania donovani* lipophosphoglycan causes periphagosomal actin accumulation: Correlation with impaired translocation of PKCalpha and defective phagosome maturation. Cell. Microbiol..

[101] Kumar G.A., Karmakar J., Mandal C., Chattopadhyay A. (2019). *Leishmania donovani* internalizes into host cells via caveolin-mediated endocytosis. Sci. Rep..

[102] Matte C., Casgrain P.-A., Séguin O., Moradin N., Hong W.J., Descoteaux A. (2016). *Leishmania major* promastigotes evade LC3-associated phagocytosis through the action of GP63. PLoS Pathog..

[103] Paixão A.R., Dias B.R.S., Palma L.C., Tavares N.M., Brodskyn C.I., de Menezes J.P.B., Veras P.S.T. (2021). Investigating the phagocytosis of *Leishmania* using confocal microscopy. J. Vis. Exp..

[104] Azevedo E., Oliveira L.T., Castro Lima A.K., Terra R., Dutra P.M.L., Salerno V.P. (2012). Interactions between *Leishmania braziliensis* and macrophages are dependent on the cytoskeleton and myosin Va. J. Parasitol. Res..

[105] Young J., Kima P.E. (2019). The *Leishmania* parasitophorous vacuole membrane at the parasite-host interface. Yale. J. Biol. Med..

[106] Canton J., Ndjamen B., Hatsuzawa K., Kima P.E. (2012). Disruption of the fusion of *Leishmania* parasitophorous vacuoles with ER vesicles results in the control of the infection. Cell. Microbiol..

[107] Antoine J.C., Lang T., Prina E., Courret N., Hellio R. (1999). H-2M molecules, like MHC class II molecules, are targeted to parasitophorous vacuoles of *Leishmania*-infected macrophages and internalized by amastigotes of *L. amazonensis* and *L. mexicana*. J. Cell Sci..

[108] Ndjamen B., Kang B.-H., Hatsuzawa K., Kima P.E. (2010). *Leishmania* parasitophorous vacuoles interact continuously with the host cell’s endoplasmic reticulum; parasitophorous vacuoles are hybrid compartments. Cell. Microbiol..

[109] Antoine J.C., Prina E., Lang T., Courret N. (1998). The biogenesis and properties of the parasitophorous vacuoles that harbour *Leishmania* in murine macrophages. Trends Microbiol..

[110] Okuda K., Tong M., Dempsey B., Moore K.J., Gazzinelli R.T., Silverman N. (2016). *Leishmania amazonensis* engages CD36 to drive parasitophorous vacuole maturation. PLoS Pathog..

[111] Batista M.F., Nájera C.A., Meneghelli I., Bahia D. (2020). The parasitic intracellular lifestyle of trypanosomatids: Parasitophorous vacuole development and survival. Front. Cell Dev. Biol..

[112] Martínez-López M., Soto M., Iborra S., Sancho D. (2018). *Leishmania* hijacks myeloid cells for immune escape. Front. Microbiol..

[113] Matte C., Arango Duque G., Descoteaux A. (2021). *Leishmania donovani* metacyclic promastigotes impair phagosome properties in inflammatory monocytes. Infect. Immun..

[114] da Silva M.F.L., Zampieri R.A., Muxel S.M., Beverley S.M., Floeter-Winter L.M. (2012). *Leishmania amazonensis* arginase compartmentalization in the glycosome is important for parasite infectivity. PLoS ONE.

[115] Boitz J.M., Gilroy C.A., Olenyik T.D., Paradis D., Perdeh J., Dearman K., Davis M.J., Yates P.A., Li Y., Riscoe M.K. (2017). Arginase is essential for survival of *Leishmania donovani* promastigotes but not intracellular amastigotes. Infect. Immun..

[116] Bichiou H., Bouabid C., Rabhi I., Guizani-Tabbane L. (2021). Transcription factors interplay orchestrates the immune-metabolic response of *Leishmania* infected macrophages. Front. Cell. Infect. Microbiol..

[117] Torrecilhas A.C., Soares R.P., Schenkman S., Fernández-Prada C., Olivier M. (2020). Extracellular vesicles in trypanosomatids: Host cell communication. Front. Cell. Infect. Microbiol..

[118] Liévin-Le Moal V., Loiseau P.M. (2016). *Leishmania* hijacking of the macrophage intracellular compartments. FEBS J..

[119] Wanderley J.L., Moreira M.E., Benjamin A., Bonomo A.C., Barcinski M.A. (2006). Mimicry of apoptotic cells by exposing phosphatidylserine participates in the establishment of amastigotes of *Leishmania (L) amazonensis* in mammalian hosts. J. Immunol..

[120] Wanderley J.L.M., Deolindo P., Carlsen E., Portugal A.B., DaMatta R.A., Barcinski M.A., Soong L. (2019). CD4^+^ T cell-dependent macrophage activation modulates sustained PS exposure on intracellular amastigotes of *Leishmania amazonensis*. Front. Cell. Infect. Microbiol..

[121] Rittig M.G., Bogdan C. (2000). *Leishmania*-host-cell interaction: Complexities and alternative views. Parasitol. Today.

[122] Bidri M., Vouldoukis I., Mossalayi M.D., Debré P., Guillosson J.J., Mazier D., Arock M. (1997). Evidence for direct interaction between mast cells and *Leishmania* parasites. Parasite Immunol..

[123] Naqvi N., Srivastava R., Selvapandiyan A., Puri N. (2020). Host mast cells in leishmaniasis: Friend or foe?. Trends Parasitol..

[124] Rodríguez N.E., Wilson M.E. (2014). Eosinophils and mast cells in leishmaniasis. Immunol. Res..

[125] Hurrell B.P., Beaumann M., Heyde S., Regli I.B., Müller A.J., Tacchini-Cottier F. (2017). Frontline Science: *Leishmania mexicana* amastigotes can replicate within neutrophils. J. Leukoc. Biol..

[126] Passelli K., Billion O., Tacchini-Cottier F. (2021). The impact of neutrophil recruitment to the skin on the pathology induced by *Leishmania* infection. Front. Immunol..

[127] Desjardins M., Descoteaux A., Gordon S. (1999). Phagocytosis of *Leishmania*: Interaction with the host and intracellular trafficking. Advances in Cellular and Molecular Biology of Membranes and Organelles.

[128] Cavalcante-Costa V.S., Costa-Reginaldo M., Queiroz-Oliveira T., Oliveira A.C.S., Couto N.F., Dos Anjos D.O., Lima-Santos J., Andrade L.O., Horta M.F., Castro-Gomes T. (2019). *Leishmania amazonensis* hijacks host cell lysosomes involved in plasma membrane repair to induce invasion in fibroblasts. J. Cell Sci..

[129] Silva M. (2012). Classical labeling of bacterial pathogens according to their lifestyle in the host: Inconsistencies and alternatives. Front. Microbiol..

[130] Pacakova L., Harant K., Volf P., Lestinova T. (2021). Three types of *Leishmania mexicana* amastigotes: Proteome comparison by quantitative proteomic analysis.

[131] Holzer T.R., McMaster W.R., Forney J.D. (2006). Expression profiling by whole-genome interspecies microarray hybridization reveals differential gene expression in procyclic promastigotes, lesion-derived amastigotes, and axenic amastigotes in *Leishmania mexicana*. Mol. Biochem. Parasitol..

